# A Note on the After-Treatment of So-Called “Frost-Bite”

**Published:** 1918-02

**Authors:** 


### *A Note on the After-Treatment of So-Called "Frost-Bite".*

By Frederick A. JOHNS, Harrogate. Abs. from the *Brit. Jour. Sur.*, Oct. 1916, p. 336.

Cases of trench feet seen in England after removal from France, may be classed as follows :

1. Painful feet without ulceration. The skin and underlying tissues are hyperesthetic.

2. Painful feet with loss of superficial sensation, but sensitive to deep pressure.

3. Feet with definite neuropathic changes, leading to paresis of the muscles and arthritic alterations.

The author believes the condition of trench feet due to pressure upon an anesthetic foot or leg. In the case of genuine frost-bite, the anesthesia is due either to cold and wet, or pressure, or the two conditions combined. Pressure may be due to shrinkage of shoes, puttees, etc., or perhaps to the practice of wearing two pairs of socks in shoes intended for only one. The circulatory interference thus produced causes localized gangrene. The effect is analagous to musculospiral paresis resulting from prolonged pressure on the musculospiral nerve in persons unconscious from anesthesia or alcoholic excess.

*Treatment.* In the cases of painful feet, it has been found best to leave the patient's feet free from bedclothes, either merely covered by a cradle left open at the end, or treated by the application of iced cloths, or such evaporating lotions as a weak solution of menthol in alcohol, painted on the feet. Ulcerated parts must be avoided. Thus relief is afforded and morphia is seldom necessary. When the extreme tenderness has passed, gentle massage and heat in the form of Berthollet baths (a local steam-bath) or hot-air baths, have been found beneficial. The receptical of the steam-bath should be heated to 120° F. or as near it as the patient can bear. The dry heat of electrically produced hot-air baths can be borne at a much higher degree, usually about 360°.

In cases where there is more or less anesthesia, gentle massage for from 10 to 25 minutes daily combined with Berthollet baths gives good results.

Cases with neuropathic changes have to be treated like regular neuritis. Vigorous massage is helpful if the patient can stand it. Bergonié treatment and thermo-penetration are useful. The former is a method of electrically-produced muscular exercise by suitably covered electrodes : the latter consists in passing a current of from 2,000 to 3,000 volts and from 0.5 to 3 ampères through the limb thus raising the temperature and, what is more important, producing hyperemia.

